# Supplementary material for: Temperature effects on carbon storage are controlled by soil stabilisation capacities
Source: Nat Commun. 2021 Nov 18;12:6713. doi: 10.1038/s41467-021-27101-1 (PMC8602258; doi:10.1038/s41467-021-27101-1)
Supplement: Supplementary file 1 — Supplementary Information [file 41467_2021_27101_MOESM1_ESM.pdf]

Supplementary Information

Temperature effects on carbon storage are controlled by soil stabilisation capacities

Iain P. Hartley, Tim C. Hill, Sarah E. Chadburn, Gustaf Hugelius

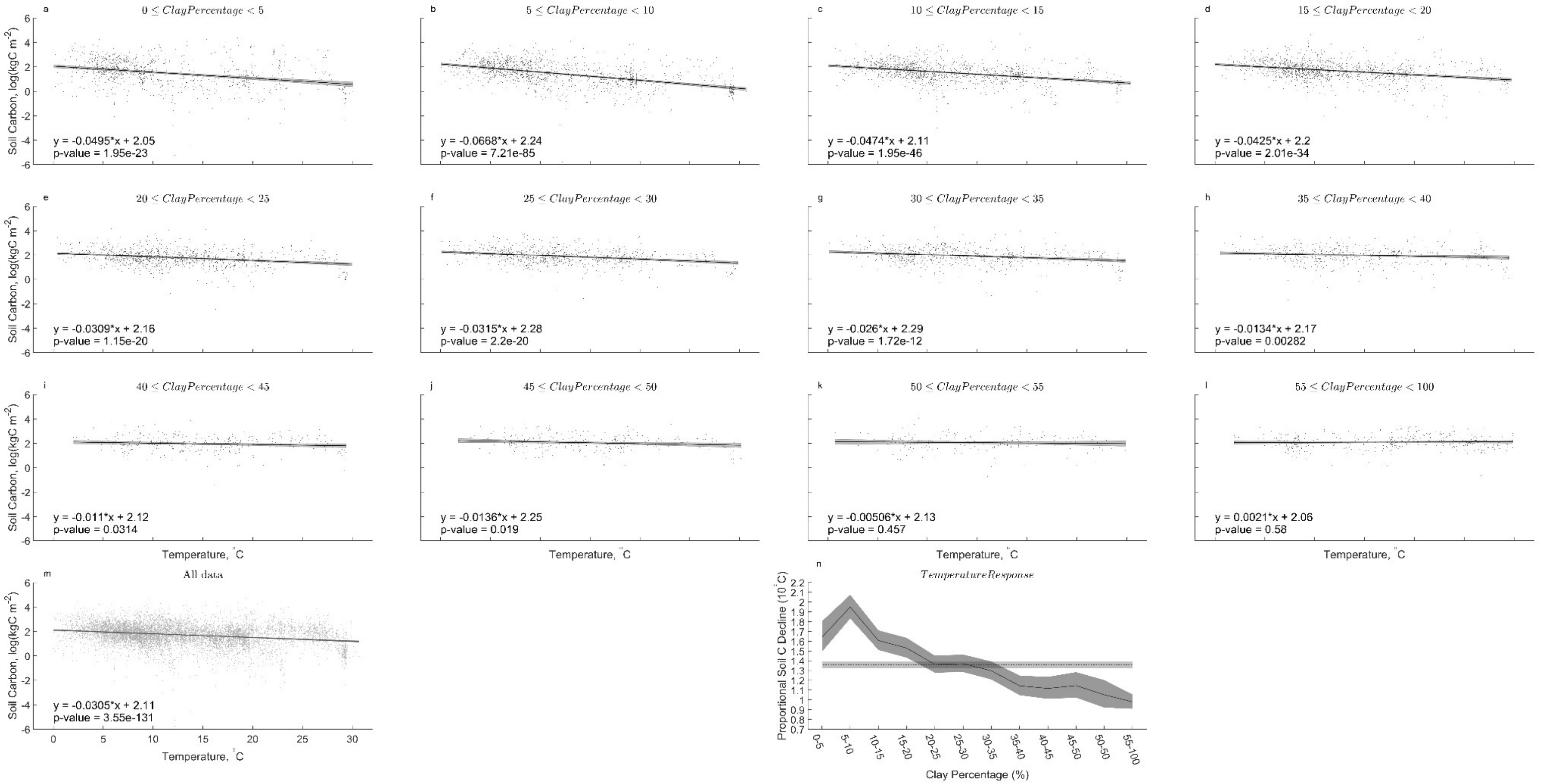

Supplementary Fig. 1 The effect of texture on the relationships between C storage in the top 50 cm of mineral soil and mean annual temperature. The relationships between temperature and soil C storage in soil with different clay contents (panels a-l represent increasing clay contents in 5% bins, but with all soils with clay contents above 55% combined), and the whole dataset (panel m). In panel n, the slopes of the relationships (solid line), together with their 95% confidence intervals (dark grey shaded area), are presented for each of the textural categories (see panels a-l) together with the slope and 95% confidence interval for the full dataset (dashed line and light grey shaded areas, see panel m).

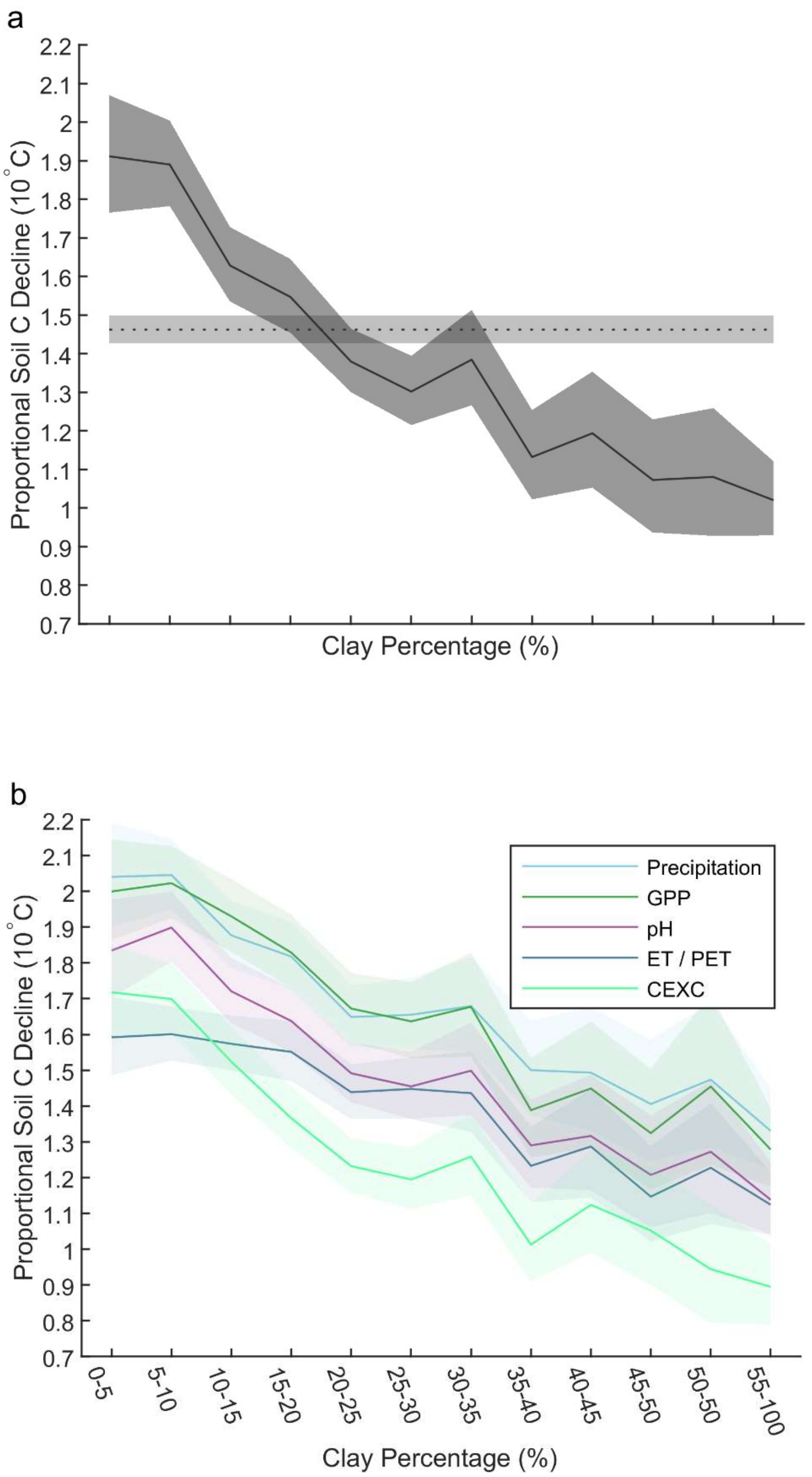

Supplementary Fig. 2 The effect of texture on the relationships between C storage in the top 20 cm of mineral soil and mean annual temperature in the raw data (panel a), and after accounting for potential confounding variables (panel b). The y-axes display the proportional reduction in C storage for each 10 °C increase in mean annual temperature, with higher values

indicating greater reductions in soil C with temperature. In panel a, the slopes of the relationships (solid line), together with their 95% confidence intervals (dark grey shaded area), are presented for each of the textural categories, with the slope and 95% confidence interval for the full dataset (dotted line and light grey shaded areas) also presented across each graph for comparison. In panel b, the relationship between soil C storage and temperature after accounting for variation in precipitation (light blue), gross primary productivity (GPP; dark green), soil pH (purple), aridity (ET / PET evapotranspiration minus potential evapotranspiration; navy blue), and cation exchange capacity (CEXC, light green) are shown. The slopes of these relationships (solid lines) together with their 95% confidence intervals (shaded area) are presented for each of the textural categories.
